# Associations of single and multiple vitamin levels with pediatric oral mucosal diseases: a cross-sectional study with multi-model analysis

**DOI:** 10.3389/fnut.2025.1677164

**Published:** 2025-11-12

**Authors:** Panpan Fang, Yingyuan Wang, Nan Chen, Kaijie Gao, Liu Yang, Xuchen Wang, Ci Li, Qianqian Sun, Tiewei Li, Junmei Yang

**Affiliations:** 1 Zhengzhou Key Laboratory of Children’s Infection and Immunity, Department of Clinical Laboratory, Henan Children’s Hospital, Zhengzhou Children’s Hospital, Children’s Hospital Affiliated to Zhengzhou University, Zhengzhou, China; 2 Department of Neonatal Intensive Care Unit, Henan Children’s Hospital, Zhengzhou Children’s Hospital, Children’s Hospital Affiliated to Zhengzhou University, Zhengzhou, China

**Keywords:** oral mucosal diseases, vitamin, vitamin mixtures, early childhood, school-age children

## Abstract

**Background:**

Vitamins play a crucial role in children’s oral health, yet the associations between multiple vitamins and pediatric oral mucosal diseases (OMDs) remain unclear. Existing studies often focus on single vitamin, leaving gaps in understanding the complex interactions of vitamin mixtures on OMDs and age-specific effects.

**Methods:**

This cross-sectional study included 1,287 children from the Children’s Hospital Affiliated to Zhengzhou University (January 2022 to April 2025), comprising 167 OMDs patients and 1,120 healthy controls. Participants were stratified into early childhood (0–6 years; *n* = 665) and school-age (6–12 years; *n* = 622) groups. Serum levels of vitamins A, D, E, C, B6, and B9 were measured. Individual and mixture effects on OMDs were assessed using multivariable logistic regression, quantile g-computation (qgComp), and Bayesian kernel machine regression (BKMR), with age-stratified analyses.

**Results:**

Our analyses consistently revealed a significant inverse association between vitamin mixtures and OMDs prevalence across all age groups (*p* < 0.05). Vitamins D, E, and B6 were significantly lower in OMDs patients versus controls (all *p* < 0.001). All models confirmed a protective association between vitamin B6 and OMDs risk. BKMR further identified a U-shaped relationship: moderate concentrations were protective, while higher levels increased risk. Quartile analysis supported this trend, with strongest protection at mid-range concentrations (Q3: OR = 0.27, 95%CI 0.15–0.45; P for trend <0.001).

**Conclusion:**

This study reveals that vitamin mixtures reduce OMDs risk in children. Vitamin B6 exhibited a U-shaped relationship, protective at moderate levels. Age-specific effects were observed: vitamin E inversely and vitamin B9 positively associated with OMDs exclusively in early childhood.

## 1 Introduction

Oral mucosal diseases (OMDs) are the third most prevalent oral health conditions, following dental caries and periodontal diseases (1). Common pediatric OMDs include recurrent aphthous stomatitis, oral lichen planus, oral candidiasis, herpetic gingivostomatitis, traumatic ulcers, cheilitis and geographic tongue (2, 3), with diverse etiological factors spanning infections, trauma, and other contributors (4). Epidemiological data reveal significant geographical variations in OMDs prevalence worldwide, ranging from 4.1 to 52.6% worldwide (5). Notably, a Chilean epidemiological study reported an exceptionally high OMDs prevalence of 30% in children (6), while a French review indicated that over one-third of school-aged children experience oral ulcers (7). In India, OMDs were found in up to 68% of children, predominantly traumatic ulcers (8). In contrast, NHANES III data from the U.S. showed a prevalence of 9.11% among children aged 2–17 years (9), highlighting considerable regional differences. Beyond clinical symptoms such as pain and difficulty eating (10), OMDs can hinder growth and development and negatively impact psychological well-being. Given their high prevalence and significant healthcare burden among pediatric populations, OMDs increase caregiving responsibilities and medical costs, representing a notable public health challenge (2, 11).

Recent years have seen growing interest in the relationship between micronutrients and oral health. As essential micronutrients for human physiology, vitamins play pivotal roles in maintaining oral mucosal integrity, modulating local immune responses, and providing antioxidant defense (12). Existing evidence indicates that vitamin A (VA) is involved in oral epithelial cell differentiation, with deficiency potentially causing abnormal mucosal keratinization (13); both vitamin D (VD) and B-complex vitamin deficiencies are strongly linked to recurrent aphthous ulcer development (13, 14); vitamin C (VC) contributes to mucosal barrier function through collagen synthesis, with studies showing lower serum levels in children with OMDs compared to healthy controls (15); although vitamin E (VE)'s role in oral health is less clearly defined, some studies have found reduced levels in patients with oral lichen planus (16). However, current research has significant limitations: most studies focus on individual vitamins, overlooking potential synergistic or antagonistic interactions among multiple vitamins, and existing data are largely derived from adult or specialized populations (e.g., diabetics), with insufficient research in pediatric cohorts. Further studies are urgently needed to explore the relationship between multiple vitamin levels and OMDs across different pediatric age groups.

Current evidence regarding the link between vitamins and pediatric OMDs is limited and inconclusive, with the precise mechanisms yet to be fully understood. In response, this study aims to use multiple statistical models, including logistic regression, qgComp, and BKMR analyses, to preliminarily examine associations between serum vitamin profiles and OMDs in children, with a particular focus on age-stratified variations in these nutrient-disease relationships. The results are expected to provide evidence-based support for nutritional interventions targeting pediatric oral mucosal disorders and offer a theoretical foundation for developing personalized prevention strategies.

## 2 Materials and methods

### 2.1 Study population

**Figure 1:**
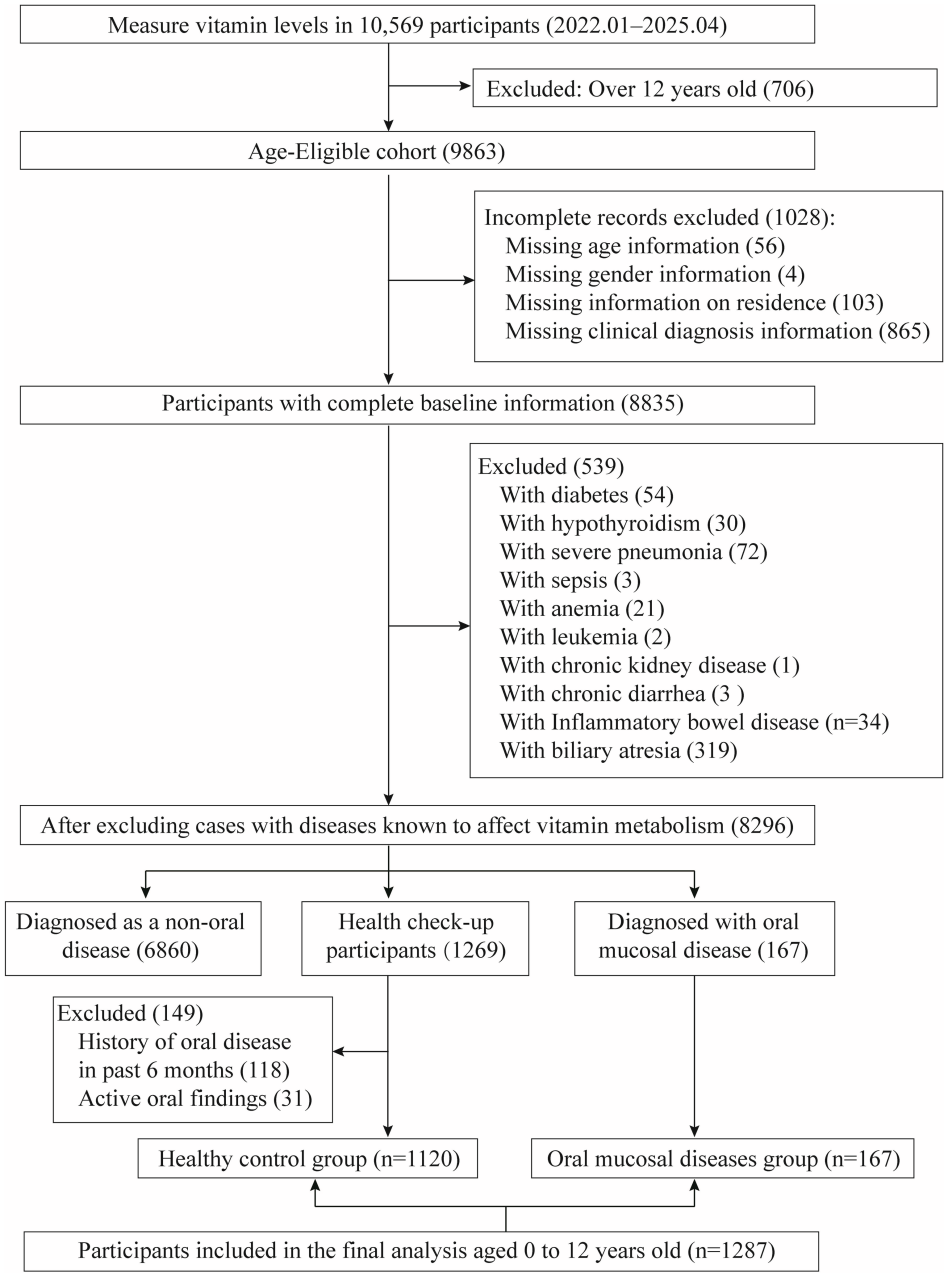
Participant selection flowchart for final analytical cohort (*n* = 1,287).

The retrospective case–control analysis was performed at the Children’s Hospital Affiliated to Zhengzhou University. We reviewed medical records of pediatric patients (*n* = 10,569) who underwent vitamin testing between January 2022 and April 2025. After applying stringent inclusion criteria, 1,287 subjects were eligible for final analysis, comprising 167 cases with OMDs and 1,120 healthy controls from routine health examinations. Diagnosis of OMDs (Recurrent oral aphthous ulcers, cheilitis, etc.) was confirmed by pediatric dentists using World Health Organization (WHO) (17, 18) and the clinical guidelines of the American Academy of Pediatric Dentistry (AAPD) (19). All case records were extracted from the clinical database and underwent secondary verification by our research team to ensure rigorous and consistent case selection. Exclusion criteria comprised: (1) lack of essential demographic data (e.g., missing age, sex, or residential information); and (2) presence of underlying conditions known to affect vitamin metabolism, such as diabetes mellitus, hypothyroidism, cholestatic hepatitis, biliary atresia, and severe infectious diseases. Healthy control subjects were selected from children undergoing routine health examinations after applying the aforementioned exclusion criteria, with the following additional criteria applied: (1) no diagnosis of OMDs (either currently or within the past 6 months), as verified through medical records and health questionnaires; (2) free from oral symptoms or lesions (e.g., pain, ulcers, bleeding) at examination. Consequently, the study included 1,287 participants in the final analysis, with the selection process detailed in Figure 1.

### 2.2 Evaluation of vitamin levels

Serum levels of VA, VD, VE, VC, vitamin B6(VB6) and vitamin B9(VB9) in serum were analyzed using Ultra-Performance Liquid Chromatography-Electrospray Ionization-Tandem Mass Spectrometry (UPLC-ESI-MS/MS) with a Waters Xevo TQ-S micro system (Milford, MA, USA). Sample preparation followed according to the manufacturer’s protocol provided in the commercial assay kit (Kehua Bioengineering, Shanghai, China). All procedures adhered strictly to standardized operating protocols to ensure analytical accuracy and reproducibility. For analytes with undetectable levels, data imputation was performed using LOD/2. Vitamin reference values were soured from established literature (20, 21) and Mayo Clinic laboratory standards, with detailed detection limits listed in Supplementary Table S1.

### 2.3 Definition of age group

The limit of 6 years aligns with the transition to permanent dentition and formal schooling in most global health frameworks (22, 23). Based on standard developmental benchmarks, the pediatric age groups were defined as follows: (1) Early Childhood (0–6 years): A critical stage for oral and systemic development, including primary tooth eruption, immune system maturation, and rapid growth; (2) School Age (6–12 years): A transitional period marked by mixed dentition (from primary to permanent teeth) and increased dietary autonomy, which impacts both nutritional status and OMDs risk.

### 2.4 Data acquisition

A retrospective medical record review was conducted utilizing the electronic medical records (EMR) system of our hospital. Clinical characteristics and laboratory test results were collected for both the healthy control group and the OMDs group. The extracted data encompassed the following: (1) demographic information: including sex, age and residence; (2) serum vitamin levels: VA, VD, VE, VC, VB6, and VB9 (folate), these measurements were performed based on clinical necessity and parental request, as assessed by healthcare providers. All vitamin assays were conducted in accordance with standardized clinical protocols, and the results were documented in the EMR for subsequent analysis.

### 2.5 Statistical approach

Categorical variables were summarized using frequency distributions (*n*, %) and compared across groups with chi-square tests. The normality of continuous variables was assessed using the Shapiro–Wilk test. Normally distributed data are presented as mean ± SD and analyzed using Student’s t-test, while non-normally distributed data are expressed as median (IQR) and analyzed using the Mann–Whitney U test. Inter-vitamin correlations were examined using Spearman’s method.

#### 2.5.1 Regression model

To evaluate associations between serum vitamin concentrations and OMDs, we performed multivariable logistic regression analyses. Given the potential non-linear relationship of these associations, vitamin levels were quartile-stratified (Q1-Q4), with Q1 serving as the reference group. Three tiered analytical models were sequentially established to evaluate robustness and to adjust for potential confounders: Model I: Adjusted for demographic variables, including sex, age, and residence. Model II: Additionally adjusted for all other vitamins (A, D, E, C, B6, B9) to account for potential inter-nutrient interactions. The results are presented as adjusted odds ratios (ORs) and their 95% confidence intervals (CIs), comparing higher quartiles (Q2–Q4) against the lowest quartile (Q1).

#### 2.5.2 Quantile G-computation (qgComp) model

To evaluate the combined and individual effects of vitamin mixtures on the risk of OMDs, we employed qgComp, a robust statistical approach that addresses limitations of traditional weighted quantile sum (WQS) regression (24). This method constructs a weighted index by assigning directional weights to each vitamin component, thereby resolving the issue of directional homogeneity (i.e., accounting for both positive and negative effects within a mixture). All vitamin concentrations (*A, D, E, C, B6, B9*) were transformed into quartiles to ensure comparability. The model estimated adjusted ORs (95% CIs) for OMDs per simultaneous one-quartile increase across all vitamins. Uncertainty estimates were derived using 1,000 bootstrap samples to ensure robustness. Positive weights indicate a risk-increasing effect, while negative weights suggest a protective effect of individual vitamins within the mixture.

#### 2.5.3 Bayesian kernel machine regression (BKMR) model

To investigate potential nonlinear and non-additive interactions among vitamins and to complement the qgComp analysis, we implemented BKMR (25). The analysis was performed using the kmbayes package with 1,000 training iterations, incorporating variable selection (varsel = TRUE) for model refinement. The BKMR analysis focused on three key exposure-response relationships: (1) The overall effect of the vitamin mixture on OMDs risk, with all vitamin concentrations fixed at their median values; (2) Vitamin-specific exposure-response relationships, with other vitamins held at their median concentrations; (3) The effect of individual vitamins across different mixture percentiles (25th, 50th, and 75th). We evaluated the association for an interquartile range (IQR) increase in each vitamin while maintaining other vitamins at specified percentiles. (4) Vitamin-vitamin interactions were assessed through stratified exposure modeling: one vitamin’s dose–response was plotted while constraining the interacting vitamin to quartile values (Q1, Q2, Q3, Q4) and stabilizing remaining vitamins at median concentrations. Posterior inclusion probabilities (PIPs) were computed to quantify the proportional influence of individual vitamins on OMDs risk.

#### 2.5.4 Age-stratified evaluation

Consistent with earlier epidemiological evidence demonstrating age-related differences in OMDs prevalence (26, 27) identified significant variations in serum concentrations of VA, VD, VE, VC, VB6, and VB9 between early childhood (0–6 years) and school-age (6–12 years) groups (Supplementary Table S2). To account for potential age-specific associations between vitamins and OMDs, we stratified all analyses by developmental stage. Specifically, we implemented a multi-model approach incorporating: (1) age-stratified mixture models (qgComp and BKMR) to evaluate differential effects of vitamin combinations; (2) separate logistic regression models for each age group. All models adjusted for sex, residence, and (where applicable) co-exposure to other vitamins, ensuring robust estimation of age-dependent effects while maintaining statistical power within each subgroup.

All statistical analyses were performed with R version 4.4.1, employing the built-in ‘stats’ package for both logistic and linear regression modeling. A two-tailed testing approach was adopted, considering results statistically significant at *p* < 0.05. The ‘qgcomp’ package facilitated the qgComp analysis, while BKMR was executed using the ‘bkmr’ package. In the context of BKMR, relationships were evaluated by computing posterior exposure effects (95% CIs) and analyzing corresponding dose–response gradients.

## 3 Results

### 3.1 Baseline characteristics

The demographic characteristics of the study population and vitamin concentration distributions are summarized in Table 1. The final analytical cohort comprised 1,287 eligible participants, with 167 individuals (12.98%) diagnosed with OMDs. The specific diagnostic distribution is as follows: recurrent aphthous stomatitis 77 (46.1%), oral candidiasis 47 (28.1%), traumatic ulcers 27 (16.2%), and other conditions 16 (9.6%) (see Supplementary Table S3). Comparative analysis revealed significantly lower serum levels of VD, VE, and VB6 in participants with OMDs compared to healthy controls (all *p* < 0.001). Correlation analysis showed weak associations among the six measured vitamins, with all Spearman’s *ρ* values < 0.55 (Supplementary Figure S1), indicating no significant multicollinearity that would affect subsequent regression analyses.

**Table 1:** Baseline participant characteristics in our study.

Characteristic	Overall	OMDs	Healthy controls	*p* value
	*N* = 1,287	*N* = 167	*N* = 1,120	
Gender, *n* (%)				0.020
Boy	781 (59.46%)	115 (69.23%)	666 (59.46%)	
Girl	506 (40.54%)	52 (30.77%)	454 (40.54%)	
Age Group, *n* (%)				<0.001
Early childhood	665 (51.51%)	59 (37.50%)	606 (54.11%)	
School Age	622 (48.49%)	108 (62.50%)	514 (45.89%)	
Residence, *n* (%)				<0.001
Urban	1,037 (80.20%)	115 (68.75%)	922 (82.32%)	
Rural	250 (19.80%)	52 (31.25%)	198 (17.68%)	
Vitamin concentrations				
VA (ng/mL), mean ± SD	339.60 ± 95.85	331.20 ± 101.28	340.85 ± 95.00	0.225
VD (ng/mL), mean ± SD	26.17 ± 10.08	22.42 ± 8.63	26.73 ± 10.17	<0.001
VE (ug/mL), mean ± SD	7.54 ± 2.30	6.66 ± 2.15	7.67 ± 2.30	<0.001
VC (ug/mL), median (IQR)	10.89 (6.91, 15.00)	10.60 (7.13, 15.11)	10.94 (6.90, 14.97)	0.848
VB6 (ng/mL), median (IQR)	7.76 (4.29, 11.75)	4.62 (2.64, 7.85)	8.20 (4.91, 12.08)	<0.001
VB9 (ng/mL), median (IQR)	14.37 (8.43, 24.76)	12.36 (7.07, 22.21)	14.71 (8.57, 24.90)	0.095

Group comparisons: *t*-test/K-S test for continuous variables; *χ*^2^ test for categorical variables; OMDs, Oral mucosal diseases; VA, vitamin A; VD, vitamin D; VE, vitamin E; VC, vitamin C; VB6, vitamin B6; VB9, vitamin B9.

### 3.2 Association between vitamins and OMDs in logistic regression model

**Figure 2:**
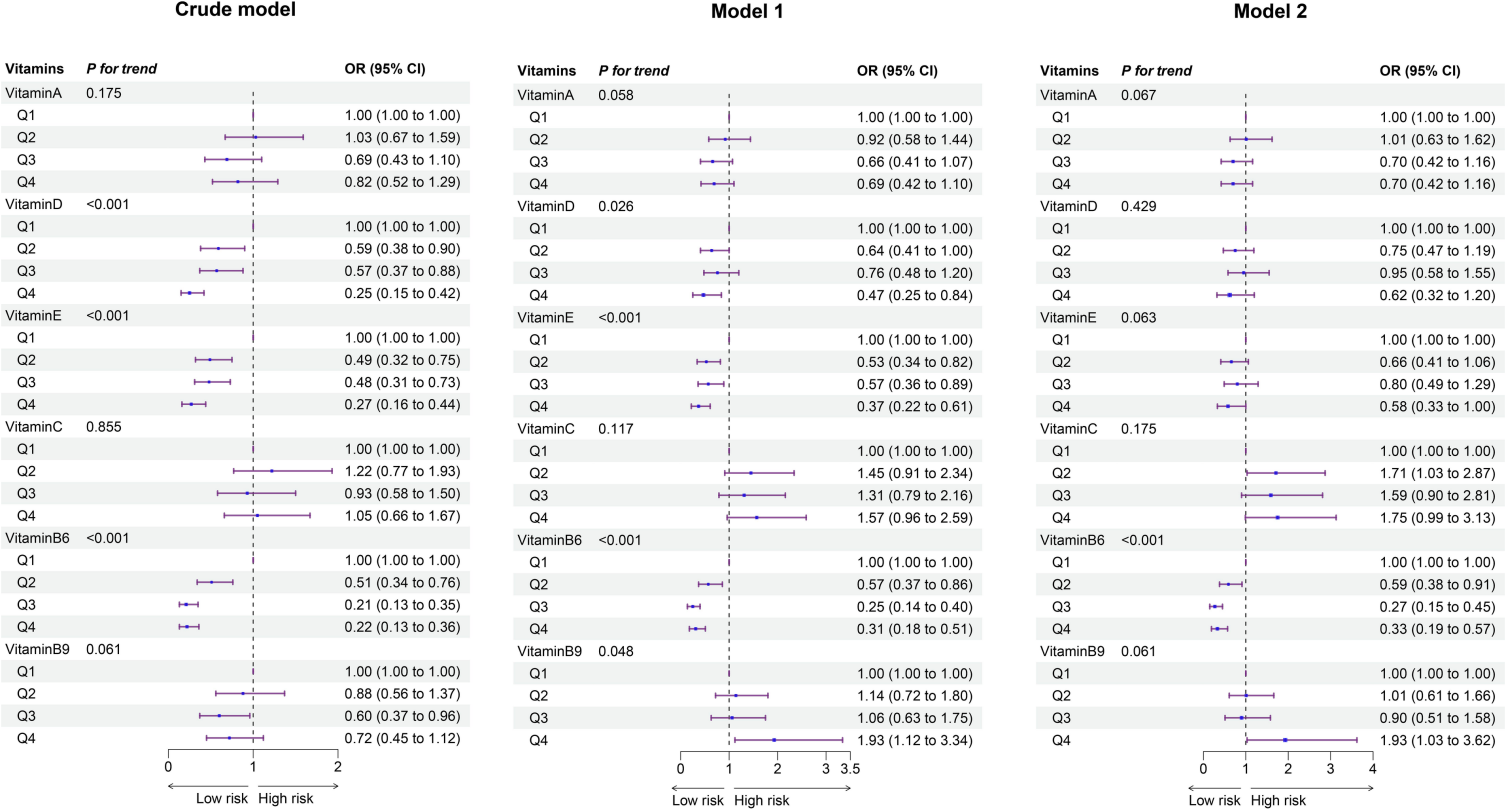
Forest plot of vitamin- OMDs associations: adjusted odds ratios from single-vitamin logistic regression models (overall population). The crude model did not adjust for any covariates. Model I accounted for age, sex, and residence. Model II included additional adjustments for all other vitamins. Ref, Reference; 95%CI, 95% confidence interval.

In the analysis of vitamin levels categorized by quartiles, three sequential logistic regression models (crude model, Model 1, and Model 2) were constructed for each individual vitamin. The results were consistent across all models, with Model 2—adjusted for the most comprehensive set of covariates—selected as the primary analytical framework (Figure 2). For the overall cohort of children aged 0–12 years, significant associations were observed between OMDs risk and VB6. Higher VB6 concentrations showed a protective dose–response trend (*p* < 0.001), with the risk of OMDs reduced in the second quartile (Q2) compared to Q1 (OR = 0.59, 95% CI: 0.38–0.91). A more substantial reduction in risk was observed in Q3 (OR = 0.27, 95% CI: 0.15–0.45), and Q4 also showed a decreased risk (OR = 0.33, 95% CI: 0.19–0.57). These findings suggest that higher VB6 levels may have a protective effect on oral mucosal health in children. Both VA (P for trend = 0.067 in Model 2) and VE (P for trend = 0.063 in Model 2) showed borderline significant protective trends. VB9 displayed a critical risk trend (P for trend = 0.061 in Model 2), with Q4 showing an increased risk (OR = 1.93, 95% CI: 1.03–3.62) compared to Q1.

Age-stratified analyses revealed distinct patterns of association between vitamin levels and OMDs risk across developmental stages. In early childhood, VB6 demonstrated a consistent protective effect, with the risk of OMDs reduced by 73% (OR = 0.27, 95% CI: 0.11–0.62) in Q3 and 80% (OR = 0.20, 95% CI: 0.08–0.48) in Q4 compared to Q1 (P for trend < 0.001). Similarly, VE showed protective associations (P for trend = 0.029). In contrast, elevated VB9 levels emerged as a significant risk factor in this age group (P for trend = 0.007). For school-aged children, VB6 retained its protective role, with risk reductions of 48% (OR = 0.52, 95% CI: 0.30–0.90) in Q2, 81% (OR = 0.19, 95% CI: 0.08–0.38) in Q3, and 58% (OR = 0.42, 95% CI: 0.20–0.83) in Q4 compared to Q1 (P for trend < 0.001). No significant associations were observed between OMDs risk and VA, VD, VE, VC, or VB9 in school-aged children (Supplementary Figures S2 and S3).

### 3.3 The vitamins with risk of OMDs in the qgComp model

**Figure 3:**
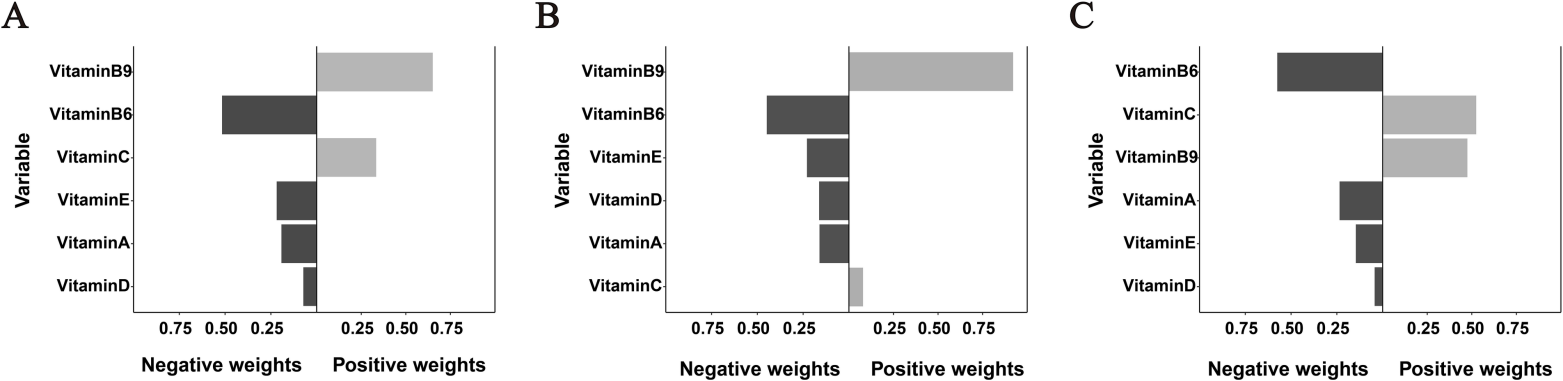
Weighted contributions of individual vitamins to OMDs risk derived from qgComp analysis in **(A)** the overall population, **(B)** early childhood, and **(C)** school-age children. All models were adjusted for age, sex, residence, and co-exposure to other vitamins.

The qgComp model analysis revealed significant associations between the combined vitamin mixture and OMDs risk across study populations (Supplementary Table S4; Figure 3). For the overall cohort, each simultaneous quartile increase in all six vitamins was associated with a 51% reduction in the odds of OMDs (OR = −0.51, 95% CI: −0.83, −0.19). A stronger protective association was observed in early childhood (OR = −0.73, 95% CI: −1.27, −0.19) compared to school-aged children (OR = −0.40, 95% CI: −0.78, −0.03). Weight analysis of individual vitamin contributions showed consistent negative weights for VA, VD, VE, and VB6 across all populations, indicating protective effects. In contrast, VC and VB9 exhibited positive weights in the overall population, suggesting potential risk-enhancing effects when considered within the vitamin mixture context.

Bar color intensity corresponds to effect magnitude, with darker shades on the negative axis (left) indicating protective associations and positive values (right) representing risk-enhancing effects. Important notes: (1) Bar lengths indicate relative within-direction effect sizes and should not be directly compared between positive and negative axes; (2) While VB9 exhibited a positive weight of 0.921 in panel B, the overall association failed to achieve statistical significance.

### 3.4 BKMR analysis of vitamin—OMDs relationships

The BKMR model analysis further elucidated both individual and combined effects of vitamins on OMDs risk, with posterior inclusion probabilities (PIPs) quantifying each vitamin’s relative importance. In the overall population, higher levels of the combined vitamins showed a protective association with OMDs. Notably, vitamins other than VA (VB6, VD, VE, VB9, and VC) made substantial contributions, with PIP values exceeding 0.7 (Supplementary Table S5). Age-stratified analyses revealed distinct patterns: among early childhood cases, all vitamins maintained high relevance (PIPs>0.8); in contrast, for school-aged children, VB6 exhibited a significantly higher PIP (0.996), indicating strong evidence for its contribution to the outcome in the mixed-exposure model. All other vitamins showed relatively weaker associations, with PIP values consistently below 0.5.

**Figure 4:**
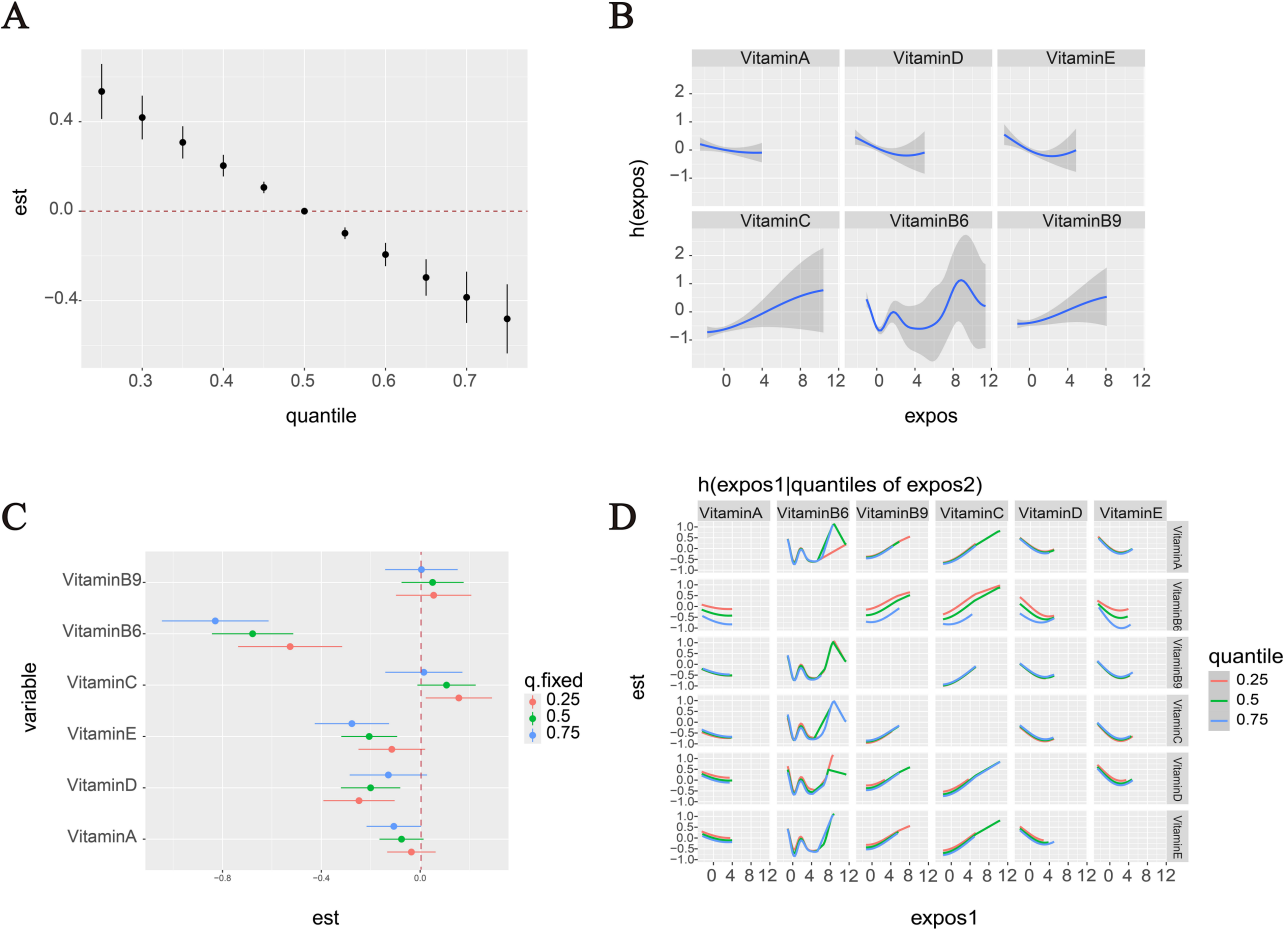
OMDs risk associations with vitamins in the overall population (BKMR analysis). **(A)** Vitamin mixtures exposure effects on OMDs risk compare to the median. **(B)** Panel **B** displays the univariate exposure-response relationships for each individual vitamin when all other vitamins are held constant at their 50th percentile concentrations. **(C)** Panel **C** specifically examines the effect of an interquartile range (IQR) increase in each vitamin while controlling for co-exposures at three distinct levels (25th, 50th, and 75th percentiles). **(D)** Two-way vitamin-OMDs associations: Column vitamins’ dose–response curves stratified by row vitamins’ exposure levels (25th/50th/75th percentiles), with other vitamins controlled at medians. All models were adjusted for potential confounders including age, sex, and residence, ensuring robust estimation of the independent and combined vitamin effects.

Figure 4 presents a comprehensive analysis of vitamin mixture effects on OMDs risk in the overall population. Panel A shows that increasing levels of the vitamin mixture in the BKMR model were significantly associated with decreased odds of OMDs. Panel B presents the univariate exposure-response relationships for individual vitamins when other vitamins were fixed at their median (50th percentile) concentrations. A nonlinear U-shaped association with OMDs risk was marginally observed for both VD and VE, with protective effects at moderate concentrations but diminished at higher levels. In contrast, VC and VB9 showed consistently positive dose–response relationships, with higher concentrations associated with increased disease risk. The associations for VB6 were less stable across its concentration ranges, exhibiting concentration-dependent variability: protective effects were observed at moderate concentrations, while the protective association attenuated or potentially reversed at higher exposure levels.

Figure 4C illustrates the estimated effects of interquartile range (IQR) increases in individual vitamin concentrations on OMDs risk, while controlling for co-exposures to other vitamins at different percentiles (25, 50, and 75%). The results revealed that serum VB6 concentrations were significantly inversely associated with OMDs risk across the total cohort. The strength of this protective association varied notably depending on the background vitamin exposure levels: when other vitamins were fixed at their 75th percentile concentrations, each IQR increase in VB6 was associated with a substantial reduction in OMDs risk (OR = −0.83, 95% CI: −1.04 to −0.61). A similar protective association persisted when other vitamins were fixed at the 50th percentile (OR = −0.68, 95% CI: −0.84 to −0.51), but the effect was attenuated at the 25th percentile (OR = −0.53, 95% CI: −0.74 to −0.32). VE and VD also showed protective associations: each IQR increase in VE was linked to reduced risk when other vitamins were fixed at the 75th and 50th percentiles (OR = −0.28, 95% CI: −0.43 to −0.13; OR = −0.21, 95% CI: −0.32 to −0.09, respectively). VD exhibited similar patterns at the 50th and 25th percentiles (OR = −0.20, 95% CI: −0.32 to −0.08; OR = −0.12, 95% CI: −0.25 to −0.02). In contrast, VC displayed positive associations with OMDs risk when other vitamins were fixed at the 25th percentile (OR = 16, 95% CI: 0.02 to 0.29).

The BKMR-based pairwise interaction analysis indicated potential interactions between high levels of VB6 and both VA and VD, as evidenced by divergent risk trends across different quantiles, suggesting that the protective effects of VB6 may be modulated by VA/VD concentrations. No significant interactions were observed for other vitamin combinations, with nearly parallel dose–response relationships (Figure 4D).

Age-stratified results (Supplementary Figures S4, S5) showed that in early childhood, increasing overall vitamin levels were significantly associated with a protective effect against OMDs (Supplementary Figures S4A and S5A). School-age children also exhibited a similar trend, with increasing vitamin concentrations correlating with reduced risk. Univariate exposure-response analysis (Supplementary Figures S4B, S5B) revealed significant age-related differences in the relationship patterns between vitamin concentrations and OMDs risk. In early childhood, VB6 demonstrated a U-shaped relationship, with decreased risk at lower concentrations, which shifted to increased risk beyond a certain threshold. VA, VD, and VE exhibited a continuous, though relatively weak, protective effect. In contrast, VC and VB9 showed a clear positive dose–response relationship, with higher concentrations corresponding to increased risk. Among school-age children, VB6 maintained its U-shaped association at low to medium concentrations, with the protective effect diminishing as concentrations increased. Notably, no significant associations were observed for VA, VD, VE, VC, or VB9 levels and OMDs risk in this age group.

A single-element analysis revealed significant age-specific correlations between vitamin exposure and OMDs (Supplementary Figures S4C and S5C). In early childhood, when co-exposure vitamins were fixed at the 25th, 50th, and 75th percentiles, serum VB6 consistently demonstrated a significant protective effect with each IQR increase (OR = −0.39, 95% CI: −0.59 to −0.19; OR = −0.42, 95% CI: −0.59 to −0.26; OR = −0.44, 95% CI: −0.63 to −0.24). Additionally, VD and VE showed protective effects against OMDs development. Conversely, VB9 was associated with an increased risk under similar conditions (other vitamins at the 25th and 50th percentiles: OR = 0.19, 95% CI: 0.01 to 0.36; OR = 0.19, 95% CI: 0.03 to 0.34). Among school-age children, the protective effect of VB6 remained significant (other vitamins at the 25th, 50th, and 75th percentiles: OR = −0.63, 95% CI: −0.93 to −0.33; OR = −0.69, 95% CI: −0.91 to −0.46; OR = −0.75, 95% CI: −1.04 to −0.45), while no statistically significant associations were observed for the other five vitamins across all exposure levels. The age-stratified analysis also revealed no significant interaction effects among the six vitamins in either early childhood or school-aged children (Supplementary Figures S4D and S5D). Specifically, the impact trends of individual vitamins on OMDs remained consistent across different exposure percentiles, without demonstrating statistically significant variations.

## 4 Discussion

Vitamin homeostasis is essential for both systemic and oral health, mediated through local biological mechanisms and systemic nutritional pathways (12, 13, 28, 29). This study employed multivariable logistic regression, qgComp, and BKMR to analyze the associations between six vitamins (A, D, E, C, B6, B9) and the risk of pediatric OMDs. A significant inverse association was observed between mixed vitamin concentrations and OMDs risk in children aged 0–12 years. BKMR analysis further suggested a U-shaped relationship between VB6 levels and OMDs risk, indicating the presence of an optimal concentration range for protective effects.

Three complementary models were used to assess the relationship between vitamins and OMDs. Logistic regression highlighted the protective effects of VB6 but masked nonlinear trends due to categorical percentile grouping. BKMR modeling revealed a U-shaped relationship for VB6, demonstrating concentration-dependent protection. Although qgComp did not detect nonlinearity, it consistently indicated an overall protective effect of the vitamin mixture, supported by BKMR’s mixture analysis (Figure 4A). These results suggest that single-vitamin analyses may misrepresent true health risks, emphasizing the importance of using multiple analytical approaches in nutritional epidemiology.

Extensive research has explored the associations between various vitamins and oral mucosal disorders. However, literature on pediatric populations remains limited compared to adults; thus, a small number of references cited here derive from adult studies. This further highlights the critical need for more studies focused on child populations. Clinical studies (30–32) have consistently shown significantly lower serum vitamin B12 levels in patients with recurrent aphthous ulcers compared to healthy controls. Substantial evidence (29, 33, 34) further supports a significant correlation between VD status and the risk of OMDs in children. A systematic review (29) highlighted the crucial preventive roles of both fat-soluble and water-soluble vitamins in pediatric oral mucosal disorders, while multiple clinical trials (29, 35) have confirmed the therapeutic potential of vitamin supplementation as an effective adjuvant treatment, underscoring the close relationship between vitamin nutritional status and oral mucosal health. Current research primarily focuses on individual vitamins, with limited exploration of the combined effects of serum vitamin mixtures in humans. Studies on single vitamins may not fully capture overall nutritional status due to the complex interactions between vitamins *in vivo*. Notably, there is a lack of dedicated investigations on the association between serum vitamin mixtures and OMDs in pediatric populations, representing a critical gap in current research that warrants further exploration.

Our findings demonstrate a significant negative correlation between serum vitamin mixture levels and the risk of OMDs. Existing evidence suggests that B-vitamin deficiency may impair oral health through various mechanisms, including compromised oral epithelial development, disrupted tooth formation, impaired enamel mineralization, and increased susceptibility to periodontitis (13, 29). Epidemiological studies consistently support the protective association between VB6 and oral mucosal health. VB6 deficiency in children is clinically manifested as angular cheilitis, glossitis, recurrent aphthous ulcers, and halitosis, with immunosuppression potentially increasing susceptibility to oral fungal infections (36, 37). However, current evidence on the association between VB6 and oral mucosal health remains limited, particularly in pediatric populations. Our study provides new insights by demonstrating significantly lower serum VB6 levels in children with OMDs compared to healthy controls. All three statistical models consistently indicated a protective effect of VB6 against OMDs across all pediatric age groups. BKMR analysis further revealed a U-shaped relationship, suggesting a potential risk associated with excessively high VB6 levels. Additionally, we observed a possible interaction between VB6 and vitamins A/D in the overall population, although this was not confirmed in age-stratified analyses, likely due to limited sample sizes in the subgroups. Future studies should focus on: (1) large-scale analyses of vitamin-vitamin interactions; (2) mechanistic research on VB6’s role in oral mucosal health; and (3) establishing pediatric-specific VB6 reference ranges for oral health. These efforts will aid in the development of nutritional strategies to prevent childhood OMDs.

Clinical evidence also indicates an inverse correlation between serum VD levels and OMDs risk in children, supporting its therapeutic potential (33, 38, 39). Our multi-model analysis further substantiates VD as a protective component of the vitamin mixture. Its unique anti-inflammatory and antimicrobial properties can significantly mitigate oral inflammatory responses (40). Notably, our age-stratified analysis revealed distinct age-specific variations in VD’s protective effects. BKMR modeling identified a weak U-shaped correlation between serum VD concentrations and OMDs risk in both the overall population and the early childhood subgroup. This biphasic relationship suggests that while moderate VD levels confer protection through immunomodulatory and antimicrobial mechanisms, both deficiency and supraphysiological concentrations paradoxically diminish these beneficial effects—a finding consistent with fundamental homeostatic principles of serum micronutrient regulation (41). The early childhood group exhibited significantly higher serum VD levels compared to the school-age group (Supplementary Table S2), with more pronounced protective effects observed in the younger population. These findings highlight the importance of VD in pediatric oral health and highlight the need for age-specific nutritional recommendations.

Previous studies (15, 34) have linked VA deficiency to oral conditions such as aphthous ulcers and cheilitis in children, while VE may serve as a diagnostic marker for such disorders (42). Our qgComp analysis revealed negative weights for both VA and VE in relation to the occurrence of OMDs (Figure 3). Age-stratified BKMR modeling further showed an inverse correlation between VA/VE levels and OMDs risk in early childhood (Supplementary Figure S4), though no such association was found in school-aged children. This discrepancy may reflect age and baseline nutritional differences, suggesting population-specific effects of these vitamins. Consequently, future studies should focus on multicenter prospective cohorts with sufficient sample sizes and consider establishing personalized nutritional assessment standards based on age and geographic factors to further clarify these relationships.

The relationship between VC and OMDs has yielded inconsistent findings. Zhou et al. demonstrated that VC deficiency correlated with increased susceptibility to geographic tongue and mild recurrent aphthous ulcers in children (15). However, Li and Zhang’s (42) study found no significant difference in serum VC concentrations between recurrent aphthous stomatitis patients and healthy controls, which aligns with our results showing no significant variation in serum VC levels between healthy controls and OMDs groups. Notably, both qgComp and BKMR analyses consistently indicated a potential positive relationship between VC levels and OMDs risk. These discrepancies may arise from variations in VC nutritional status among study populations, as the overall VC deficiency rate was relatively low in our cohort (Supplementary Table S6).

Regarding VB9 (folic acid), Wu et al.’s (43) study on Taiwanese male populations found a higher prevalence of folic acid deficiency among oral submucous fibrosis patients. Although no statistically significant differences in VB9 levels were observed between OMDs groups and healthy controls, analysis across all three models consistently identified elevated VB9 levels as a risk factor for OMDs, particularly in the early childhood group. This may be linked to the significantly higher VB9 levels found in the early childhood group compared to the school-age group (Supplementary Table S2), suggesting a potential “biphasic effect” of VB9, where optimal protective effects are seen only within a specific physiological concentration range. Currently, no clinical guidelines define an upper reference limit for serum VB9, and evidence linking VB9 to OMDs remains limited. Further high-quality studies are urgently needed to clarify this relationship.

This study offers novel insights into the association between OMDs in children and serum vitamin levels, though several limitations must be acknowledged. First, the sample was drawn from a single region, which limits the generalizability of the findings to other populations and geographic areas. Second, due to the retrospective design, potential confounding factors such as oral hygiene practices, detailed dietary intake, and socioeconomic status, which may influence the risk of OMDs, could not be controlled. These limitations constrain our ability to establish definitive causal relationships between vitamin levels and OMDs. Larger prospective studies that incorporate these critical variables are necessary to draw more conclusive findings. Our research team intends to use institutional resources to conduct a longitudinal study examining the relationship between serum vitamin levels and OMDs in children within this region, with the goal of generating stronger evidence-based medical conclusions.

## 5 Conclusion

Our study demonstrates that mixed vitamin levels are inversely associated with the risk of OMDs in children. VB6 showed a nonlinear, concentration-dependent protective relationship. Age-specific effects were also observed: VE was inversely correlated and VB9 was positively associated with OMDs risk in early childhood, although these associations were not significant in school-aged children. These findings highlight the importance of considering vitamin mixtures and age stratification in nutritional epidemiology. Maintaining adequate levels of key vitamins, particularly within optimal ranges for nutrients like VB6, may help reduce the risk of oral mucosal diseases in children. Further prospective and mechanistic studies are required to confirm these relationships and explore their clinical relevance, thereby providing scientific support for developing age-specific precision nutrition intervention strategies.

## Data Availability

The original contributions presented in the study are included in the article/Supplementary material, further inquiries can be directed to the corresponding authors.

## Author’s note

All authors confirm that this manuscript is a transparent account of the reported research. This work is related to another study by the same author group, titled “Vitamins and the Risk of *Mycoplasma Pneumoniae* Pneumonia among Children: A Cross-sectional Study,” which is currently under review at Frontiers in Immunology. Both studies are sub-analyses stemming from the same overarching research project, “A Study Evaluating Vitamin Nutritional Status in Children of Henan Region Based on HPLC-MS/MS Detection Technology,” which is the reason for sharing the same ethical approval (No. 2022-K-L045).
