# Supplementary figures and images for: Associations of single and multiple vitamin levels with pediatric oral mucosal diseases: a cross-sectional study with multi-model analysis
Source: Front Nutr. 2025 Nov 12;12:1677164. doi: 10.3389/fnut.2025.1677164 (PMC12648972; doi:10.3389/fnut.2025.1677164)

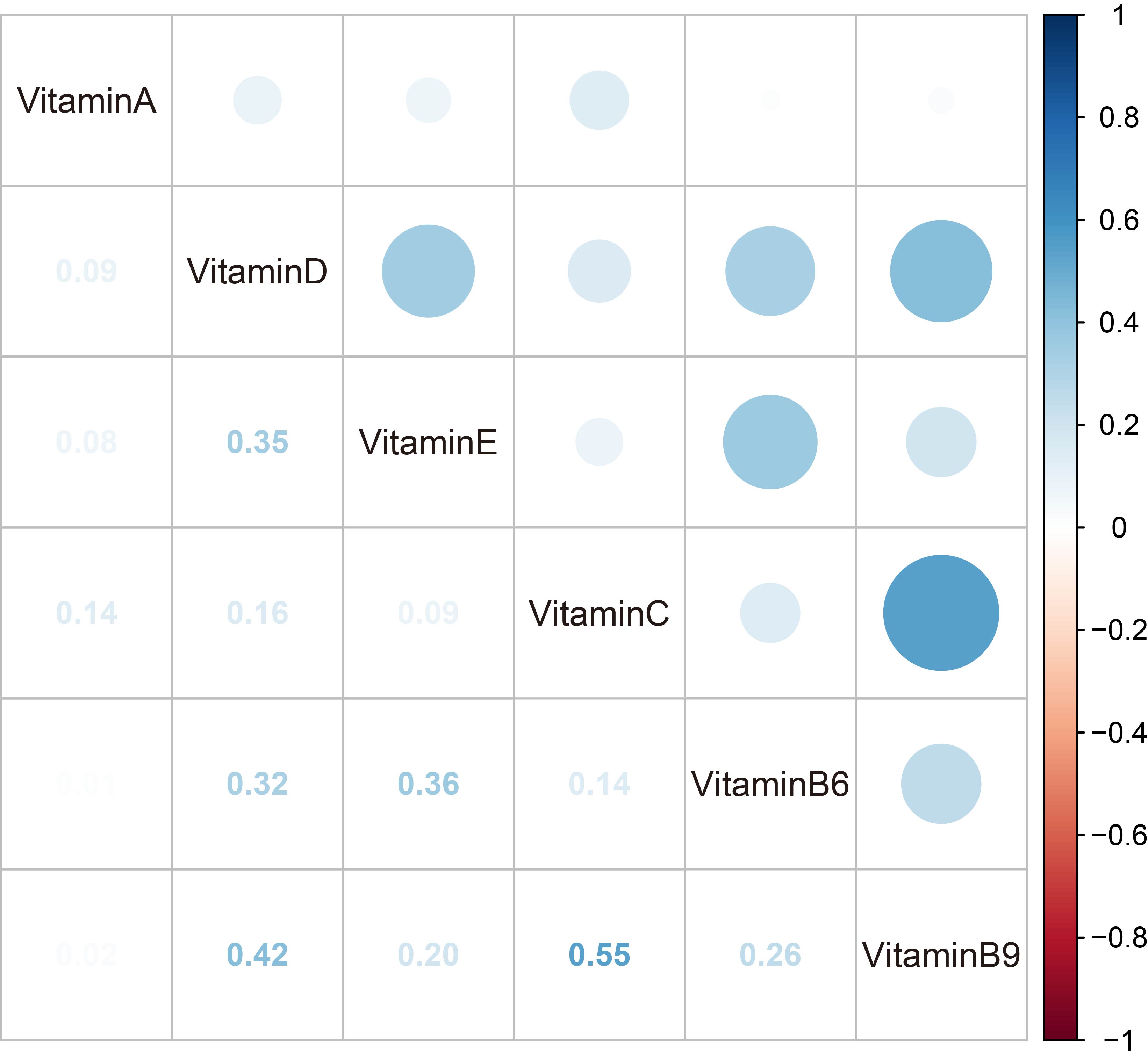

Supplement: Supplementary file 1 [file Image_1.JPEG]

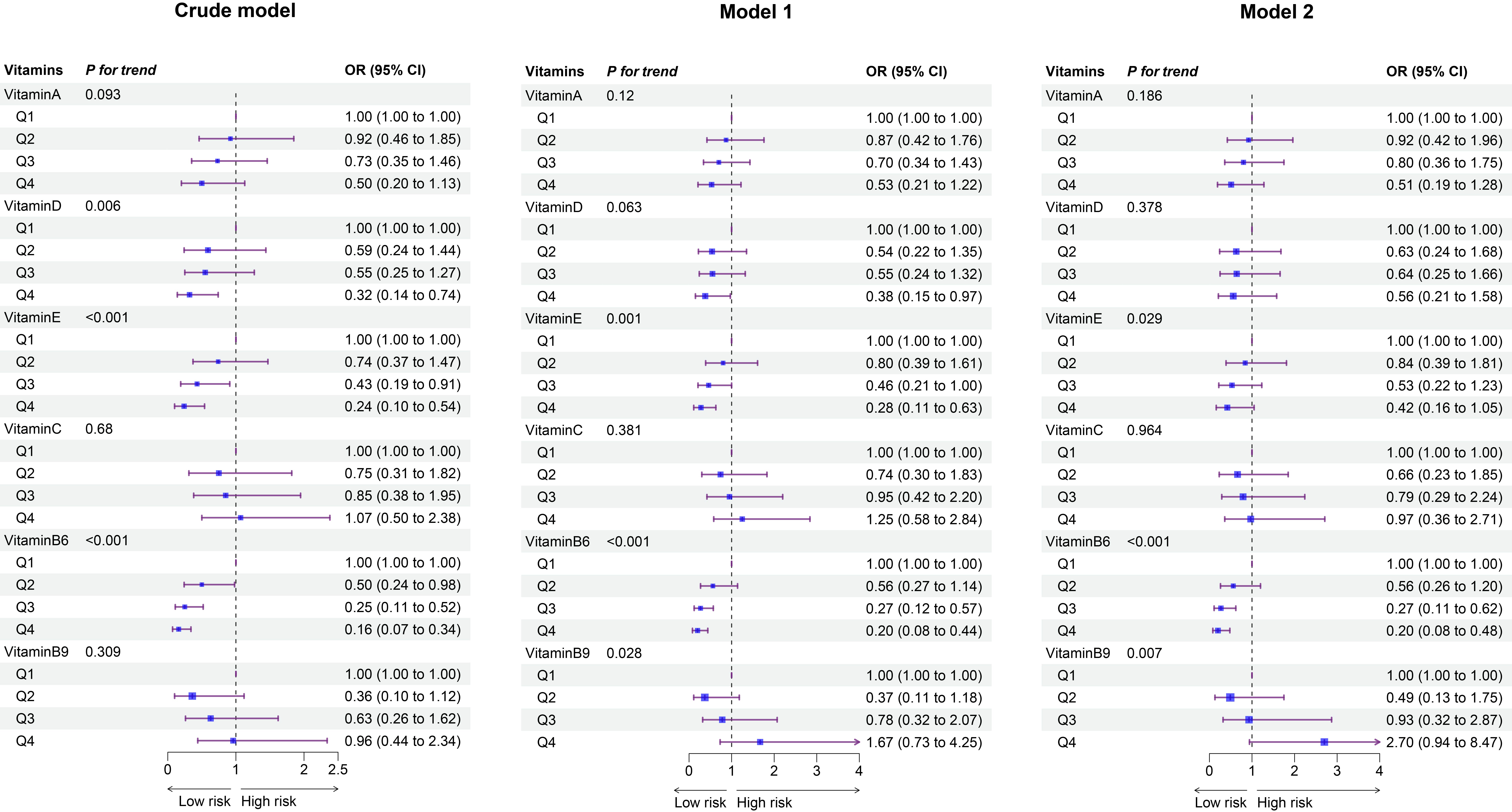

Supplement: Supplementary file 2 [file Image_2.JPEG]

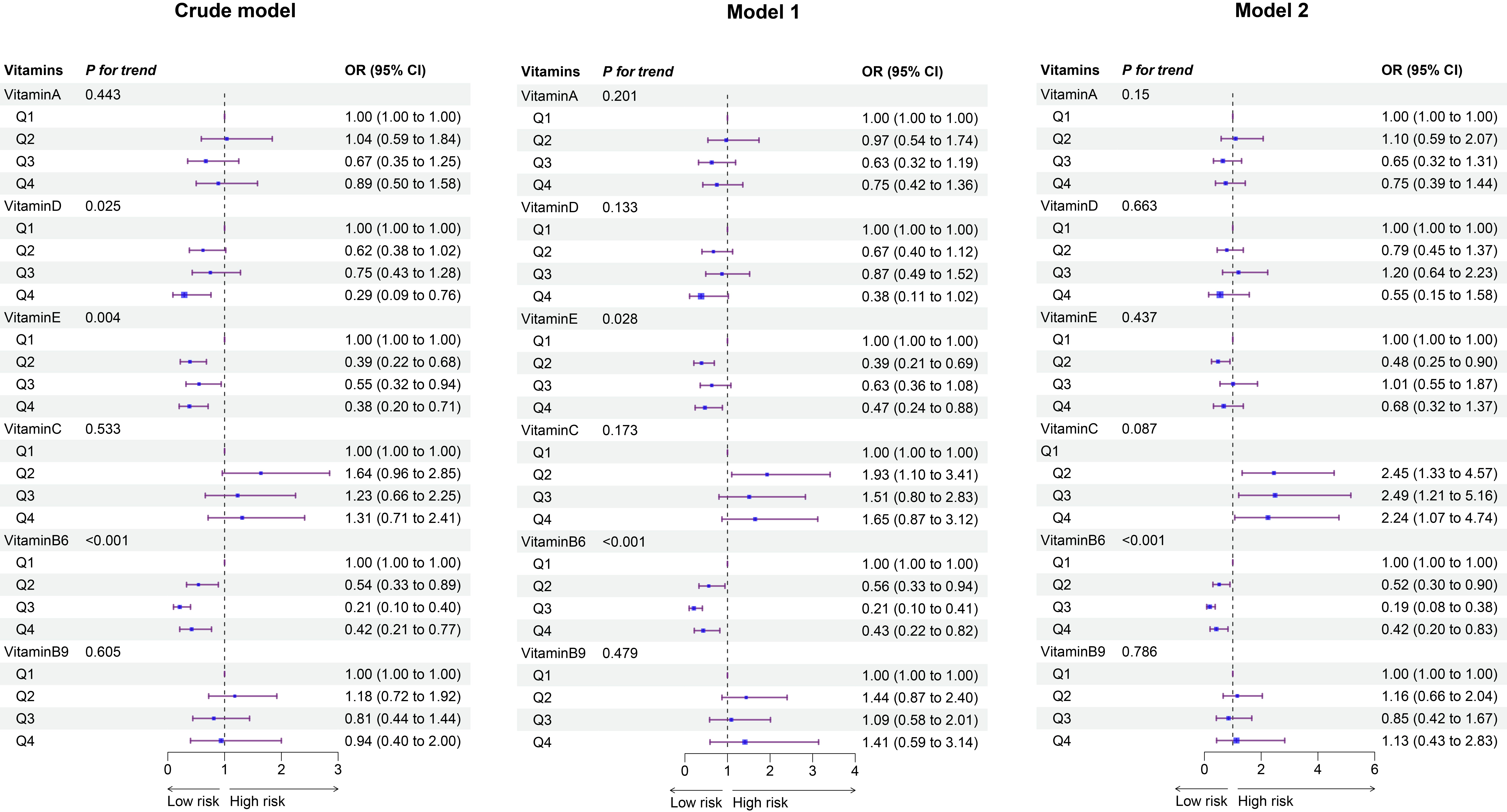

Supplement: Supplementary file 3 [file Image_3.JPEG]
