# Supplementary material for: Associations of single and multiple vitamin levels with pediatric oral mucosal diseases: a cross-sectional study with multi-model analysis
Source: Front Nutr. 2025 Nov 12;12:1677164. doi: 10.3389/fnut.2025.1677164 (PMC12648972; doi:10.3389/fnut.2025.1677164)
Supplement: Supplementary file 6 [file Table_1.DOCX]

**Supplementary Material**

**Table S1** Distributions of the vitamin concentration in our study.

**Table S2** Levels of vitamins in serum of early childhood and school-age children.

**Table S3** Distribution of OMDs in our study, n (%).

**Table S4** Association between qgComp vitamin indices and oral diseases risk.

**Table S5** PIPs for vitamin-oral disease associations derived from BKMR analysis.

**Table S6** Prevalence of vitamin deficiency in oral disease group versus healthy controls in our study.

**Figure S1** Correlational relationships among six essential vitamins.

**Figure S2** Forest plot of vitamin-oral disease associations: adjusted odds ratios from single-vitamin logistic regression models (early childhood children).

**Figure S3** Forest plot of vitamin-oral disease associations: adjusted odds ratios from single-vitamin logistic regression models (school-age children).

**Figure S4** BKMR-derived associations between serum vitamin levels and oral diseases risk in early childhood children.

**Figure S5** BKMR-derived associations between serum vitamin levels and oral diseases risk in school-age children.

**Table S1.** Distributions of the vitamin concentration in our study.

| **Vitamins** | **Biological specimens** | **Concentration** | | | **Reference value** | **Citation source** | **LLOD** | **Detection rate** |
| --- | --- | --- | --- | --- | --- | --- | --- | --- |
|  |  | **25th** | **50th** | **75th** |  |  |  |  |
| VAa | Serum | 275.13 | 327.47 | 389.3 | 200~600ng/mL | Literature | 5 | 100.00% |
| VDa | Serum | 18.96 | 25.37 | 31.95 | 20~100ng/mL | Literature | 0.25 | 100.00% |
| VEb | Serum | 5.99 | 7.3 | 8.8 | 3.8~18.4ug/mL | Literature | 0.05 | 100.00% |
| VCb | Serum | 6.91 | 10.89 | 15 | 4~20ug/mL | Literature | 0.05 | 99.77% |
| VB6a | Serum | 4.29 | 7.76 | 11.75 | 5~50 ng/mL | Literature | 0.128 | 100.00% |
| VB9a | Serum | 8.43 | 14.37 | 24.76 | >4 ng/mL | Literature | 0.128 | 99.77% |

^a^Measurement unit: ng/mL; ^b^Measurement unit: µg/mL; Abbreviations: VA, vitamin A; VD, vitamin D; VE, vitamin E; VC, vitamin C; VB6, vitamin B6; VB9, vitamin B9, LLOD, lower limit of detection.

**Table S2.** Levels of vitamins in serum of early childhood and school-age children.

| **Characteristic** | **Overall** | **Early childhood** | **School-age** | ***p* value** |
| --- | --- | --- | --- | --- |
|  | N =1287 | N = 665 | N = 622 |  |
| Vitamin concentrations |  |  |  |  |
| VAa | 339.60 ± 95.85 | 332.66 ± 94.12 | 347.02 ± 97.20 | 0.007 |
| VDa | 26.17 ± 10.08 | 30.82 ± 10.12 | 21.20 ± 7.28 | <0.001 |
| VEb | 7.54 ± 2.30 | 7.93 ± 2.42 | 7.11 ± 2.09 | <0.001 |
| VCb | 10.89 (6.91, 15.00) | 12.23 (8.12, 15.91) | 9.33 (5.53, 13.69) | <0.001 |
| VB6a | 7.76 (4.29, 11.75) | 9.03 (5.55, 13.59) | 6.34 (3.28, 9.68) | <0.001 |
| VB9a | 14.37 (8.43, 24.76) | 20.79 (13.06, 31.30) | 9.84 (6.19, 15.59) | <0.001 |

Differences in vitamins between early childhood and school-age group individuals were assessed using Student’s t-test or Mann–Whitney U test. ^a^Measurement unit: ng/mL; ^b^Measurement unit: µg/mL; Abbreviations: VA, vitamin A; VD, vitamin D; VE, vitamin E; VC, vitamin C; VB6, vitamin B6; VB9, vitamin B9.

**Table S3** Distribution of OMDs in our study, n (%).

| **Type of OMDs** | **Male** | **Female** | **Total** |
| --- | --- | --- | --- |
| Recurrent aphthous stomatitis | 19(11.38) | 58(34.73) | 77(46.11) |
| Oral candidiasis | 19(11.38) | 28(16.77) | 47(28.14) |
| Traumatic ulcers | 9(5.39) | 18(10.78) | 27(16.17) |
| Chronic non-specific cheilitis | 2(1.20) | 6(3.59) | 8(4.79) |
| Herpetic gingivostomatitis | 2(1.20) | 2(1.20) | 4(2.40) |
| Geographic tongue | 1(0.60) | 3(1.80) | 4(2.40) |

OMDs, Oral mucosal diseases.

**Table S4.** Association between qgComp vitamin indices and oral diseases risk.

| Vitamins | Overall population | Early childhood | School Age |
| --- | --- | --- | --- |
| VA | -0.193 | -0.160 | -0.235 |
| VD | -0.073 | -0.163 | -0.044 |
| VE | -0.218 | -0.229 | -0.146 |
| VC | 0.334 | 0.079 | 0.525 |
| VB6 | -0.517 | -0.448 | -0.575 |
| VB9 | 0.666 | 0.921 | 0.475 |
| Positive direction | 0.345 | 0.644 | 0.305 |
| Negative direction | -0.855 | -1.380 | -0.709 |
| Vitamin mixture OR (95%CI) | -0.510(-0.827, -0.193) | -0.731(-1.273, -0.190) | -0.404(-0.780, -0.029) |
| *P* | 0.002 | 0.008 | 0.0435 |

Data are presented as weights for individual vitamins and ORs (95% CI) for vitamin mixtures estimated using the QGC model, representing the single effect weights and combined effects of each quartile increase in vitamin mixture on the risk of oral diseases. The model was adjusted for age, sex, and residence. Abbreviations: VA, vitamin A; VD, vitamin D; VE, vitamin E; VC, vitamin C; VB6, vitamin B6; VB9, vitamin B9.

**Table S5.** PIPs for vitamin-oral disease associations derived from BKMR analysis.

| Vitamins | Overall population | Early childhood | School Age |
| --- | --- | --- | --- |
| VA | 0.478 | 0.8 | 0.412 |
| VD | 0.938 | 0.968 | 0.486 |
| VE | 0.968 | 0.884 | 0.246 |
| VC | 0.854 | 0.804 | 0.418 |
| VB6 | 1 | 1 | 0.996 |
| VB9 | 0.738 | 1 | 0.370 |

Abbreviations: PIP, posterior inclusion probability; BKMR, Bayesian kernel machine regression; VA, vitamin A; VD, vitamin D; VE, vitamin E; VC, vitamin C; VB6, vitamin B6; VB9, vitamin B9.

**Table S6.** Prevalence of vitamin deficiency in oral disease group versus healthy controls in our study.

| **Variables** | **Overall** | **Oral diseases** | **Healthy controls** | ***p* value** |
| --- | --- | --- | --- | --- |
|  | N =1287 | N = 167 | N = 1120 |  |
| VA |  |  |  |  |
| deficiency, n (%) | 56 (4.35%) | 9 (5.39%) | 47 (4.20%) | 0.481 |
| VD |  |  |  |  |
| deficiency, n (%) | 387 (30.07%) | 77 (46.11%) | 304 (27.14%) | <0.001 |
| VE |  |  |  |  |
| deficiency, n (%) | 47 (3.65%) | 12 (7.19%) | 35 (3.13%) | 0.009 |
| VC |  |  |  |  |
| deficiency, n (%) | 140 (10.88%) | 13(7.78%) | 127 (11.34%) | 0.169 |
| VB6 |  |  |  |  |
| deficiency, n (%) | 376 (29.22%) | 91 (54.49%) | 285 (25.45%) | <0.001 |
| VB9 |  |  |  |  |
| deficiency, n (%) | 86 (6.68%) | 14 ((8.38%) | 72 (6.43%) | 0.345 |

Differences in vitamin deficiency rates between the oral disease group and healthy control group were assessed using chi-square tests (or Fisher's exact tests for categorical variables with expected cell counts <5), with two-tailed P-values <0.05 considered statistically significant. Abbreviations: VA, vitamin A; VD, vitamin D; VE, vitamin E; VC, vitamin C; VB6, vitamin B6; VB9, vitamin B9.

**Figure S1.** Correlational relationships among six essential vitamins.

**Figure S2.** Forest plot of vitamin-oral disease associations: adjusted odds ratios from single-vitamin logistic regression models (early childhood children). The crude model did not adjust for any covariates. Model I accounted for age, sex, and residence. Model II included additional adjustments for all other vitamins. Abbreviations: Ref, Reference; 95%CI, 95% confidence interval.

**Figure S3.** Forest plot of vitamin-oral disease associations: adjusted odds ratios from single-vitamin logistic regression models (school-age children). The crude model did not adjust for any covariates. Model I accounted for age, sex, and residence. Model II included additional adjustments for all other vitamins. Abbreviations: Ref, Reference; 95%CI, 95% confidence interval.

**Figure S4.** BKMR-derived correlations between serum vitamin levels and oral diseases risk in early childhood children. (A) The overall effect of vitamin mixtures on oral diseases risk, shown as percentiles of increase relative to the median. (B) Panel B displays the univariate exposure-response relationships for each individual vitamin when all other vitamins are held constant at their 50th percentile concentrations. (C) Panel C specifically examines the effect of an interquartile range (IQR) increase in each vitamin while controlling for co-exposures at three distinct levels (25th, 50th, and 75th percentiles). (D) Two-way vitamin-OMD associations: Column vitamins' dose-response curves stratified by row vitamins' exposure levels (25th/50th/75th percentiles), with other vitamins controlled at medians. All models were adjusted for potential confounders including age, sex, and residence, ensuring robust estimation of the independent and combined vitamin effects.

**Figure S5.** BKMR-derived correlations between serum vitamin levels and oral diseases risk in school-age children. (A) The overall effect of vitamin mixtures on oral diseases risk, shown as percentiles of increase relative to the median. (B) Panel B displays the univariate exposure-response relationships for each individual vitamin when all other vitamins are held constant at their 50th percentile concentrations. (C) Panel C specifically examines the effect of an interquartile range (IQR) increase in each vitamin while controlling for co-exposures at three distinct levels (25th, 50th, and 75th percentiles). (D) Two-way vitamin-OMD associations: Column vitamins' dose-response curves stratified by row vitamins' exposure levels (25th/50th/75th percentiles), with other vitamins controlled at medians. All models were adjusted for potential confounders including age, sex, and residence, ensuring robust estimation of the independent and combined vitamin effects.
